# The health of working dogs in conservation in Africa

**DOI:** 10.3389/fvets.2023.1179278

**Published:** 2023-07-18

**Authors:** Nicola Earnshaw, Neil Anderson, Jill Mackay, Megan Parker

**Affiliations:** ^1^Department of Conservation Medicine, The Royal (Dick) School of Veterinary Science, The University of Edinburgh, Edinburgh, United Kingdom; ^2^Department of Veterinary Medical Education, The Royal (Dick) School of Veterinary Science, The University of Edinburgh, Edinburgh, United Kingdom; ^3^The Center for Large Landscape Conservation, Bozeman, MT, United States

**Keywords:** canine, conservation, Africa, health, thematic analysis, African trypanosomiasis, heatstroke

## Abstract

**Introduction:**

Dogs are increasingly being employed for conservation purposes worldwide. In Africa, they work in challenging environments with unique health risks which have not been investigated until now.

**Methods:**

To understand the health challenges faced by the dogs, semi-structured interviews were conducted with participants from 14 organisations that used working dogs in their conservation programmes. The data was qualitatively analysed by thematic analysis.

**Results:**

Five themes were generated. Three affective themes influenced how participants responded to the challenges associated with having a successful conservation dog programme. A strong handler-dog attachment, proficient handler training, and the acknowledgement of the challenging environment were pivotal to maintaining dog health. Two themes related to the difficulties in managing these programmes and how veterinary support interacts with the management choices being made.

**Discussion:**

To have healthy conservation dogs, current and future programmes should focus on fostering the handler-dog relationship and provide continuous handler training. The management of conservation dogs’ health should adopt an evidence-based approach. Future research should focus on areas where the evidence base is lacking, particularly in the areas of prevention and treatment of African canine trypanosomiasis. Programmes should develop a good working relationship with a veterinarian that has access to evidence-based veterinary medical information.

## 1. Introduction

The remarkable olfactory capabilities of dogs (*Canis familiaris*) combined with the ability for humans to develop close working relationships with them means that they are employed for a wide variety of tasks. Dogs are employed by conservation programs and governments worldwide for wildlife population research, monitoring, management and in preventing and enforcing wildlife crime. They are used in the detection of elusive wildlife and their scat, in invasive species detection, carcass detection and the detection of disease (1–6). In many African countries, handler-dog partnerships are used to support conservation efforts in the detection of elusive species and to track poachers from a crime scene and in detecting illegal firearms, snares and illicit wildlife trade such as ivory, pangolin scales, rhino horns and bushmeat at local roadblocks and international ports and borders (7, 8). Livestock guarding dogs are also employed by conservation organizations involved in the mitigation of human wildlife conflict (9). They play an important role in conservation, but their management is distinctly different to that of other conservation dogs which is beyond the scope of this study.

These roles require the development of complex skills on the part of both the handlers and the conservation dogs. Handlers must be skilled in reading the body language of their dogs and comfortable in the environment in which they work (10). Dogs are more accurate at detection when working with familiar handlers than with unfamiliar handlers (3, 11, 12). The dog-handler relationship is therefore an important component of the success of the program. The training of both dogs and handlers and development of the dog-handler relationship requires extensive work for the program to be successful (13).

Conservation dog partnerships are relatively under researched, with limited information regarding how dogs are sourced, trained, and partnered. The cost of investing and maintaining a program with dogs working in conservation is also unknown. Experienced trainers are required to select prospective dogs that are in good health with suitable traits and train them in their deployed location for their required task (14). In Africa, dogs are often deployed to remote locations from abroad and trained by foreign trainers, adding further to the cost of the program. Published guidelines on conservation dog selection, training and management mainly focus on the behavior and training of dogs. Very little has been published on the management of their health, other than recommending that dogs selected are healthy and of a suitable build for the environment in which they work (14, 15). However, the importance of optimizing conservation dog health cannot be underestimated. If a dog cannot carry out the work for which it is trained, then not only does it impact the welfare of the dog, but it is also the loss of an asset and loss of resource investment. In fact, canine health status is known to directly affect the olfactory capabilities of dogs upon which these programs largely rely (16).

There are very few published guidelines on the health and welfare needs of conservation dogs. A review of conservation dog programs in Africa in 2015 by the non-governmental organization Working Dogs for Conservation made recommendations for best practice for conservation dog programs, highlighting the main challenges faced from conservation dog programs, the infrastructure and the expertise that is required to establish and maintain a successful program. Recommendations that directly linked to conservation dog health included the construction of appropriate kennels and training handlers in veterinary first aid. Inadequate veterinary care was identified as one of the main challenges that conservation dog programs in Africa faced (17). The New Zealand Department of Conservation Dog/Handler Team Standard Operating Procedures gives brief guidelines on the minimum care of conservation dogs, advising adherence to the national legislation Animal Welfare (Dogs) Code 2010, vaccination and regular parasiticide treatment (18). This ensures that the health of conservation dogs is considered by those working in the industry but does not address the concerns that conservation dogs may face due to their unique circumstances.

The veterinary intervention and care required to support these partnerships is necessarily based on other working dog partnerships that are more clearly understood. Military working dogs and search and rescue dogs also perform detection and apprehension work and may have to do this in harsh terrain, facing temperature extremes and geographical isolation so are comparable to conservation dogs (19, 20). However, one must be aware of key differences between conservation dogs in Africa and other working dogs. There are possible differences in breed composition of conservation dogs compared to military working dogs. Military working dogs and search and rescue dogs often are well funded whereas conservation dog programs are often financially constrained (13). Military working dogs are deployed to challenging environments for shorter periods, whereas conservation dogs are more likely to be permanently deployed in a challenging environment for their entire working lives.

American military working dogs undergo health screening before acquisition including physical examination, hematology and serum biochemistry tests, infectious disease screening and hip and elbow screening for joint dysplasia. Female dogs are neutered at admission. Throughout their lives they undergo biannual physical examination, hematology, biochemistry and urinalysis and have regular vaccinations and parasite control. Handlers undergo basic veterinary first aid training with the opportunity for further training throughout their career (21). Similar recommendations were made by Jones et al. for search and rescue dogs (22). It was also recommended that individual programs formulate individualized veterinary health plans with the veterinarians to whom the dogs are registered and that handlers be trained in emergency first aid (22).

Examining the problems leading to loss of workdays, retirement, death and euthanasia in the military working dog and search and rescue dog literature can inform on health challenges that conservation dogs may face. Conservation dogs in Africa are likely to face similar challenges in addition to challenges unique to their specific location on the continent. A study on dogs deployed to Iraq in a 20-month period examined the reasons for 1,530 non-combat related veterinary visits in military working dogs. Dogs had dermatological problems (25%), soft tissue trauma (21%), gastrointestinal disorders (17%) and musculoskeletal disorders (14%) (21). A small survey on search and rescue dogs deployed to Haiti in response to the 12^th^ January 2010 earthquake also revealed a range of minor conditions that the dogs succumbed to, with soft tissue trauma and dehydration the most commonly identified (23). Additionally, conservation dog programs in Africa may also be challenged with tick-borne disease, canine African trypanosomiasis, snake envenomation and poisoning secondary to accidental ingestion of poisoned wildlife (24–27).

Africa was targeted for this study due to the scarcity of research into the health and welfare of conservation dogs in this region. This is of particular concern due to the valuable work that the dogs conduct combined with the challenging environments in which they are placed. In this study, we sought to characterize the health challenges faced by programs using conservation dogs in some African countries, and how they are managed, particularly around the provision of veterinary care. The overall aim in this study was to explore the difficult challenges and develop key considerations and recommendations for those deploying dogs within African countries and highlights the areas required for future research that would most benefit the welfare of conservation dogs in African programs and elsewhere.

## 2. Materials and methods

### 2.1. Position of researchers

A qualitative research approach was adopted to explore health challenges and management approaches of African conservation programs that use conservation dogs. This approach allowed the understanding of the key health challenges that conservation dogs faced and the complexities around managing such programs (28). The semi-structured qualitative research interview was used to gain rich and detailed information about the health challenges that conservation dogs face, and the methods of management adopted by the programs. This enabled health challenges and management decisions that are complex and influenced by multiple factors to be understood. It also identified the social factors that affected conservation dog health (28, 29).

Thematic analysis was used to identify patterns in the data and thus extract main themes. Themes identified from the data allowed for the perspectives of the participants to be communicated directly from their own experiences, rather than using predefined parameters (30).

The three principal leads were NEE, a white female cisgendered practicing veterinarian with experience of the working dog literature. NA is a white male cisgendered veterinarian with experience working in southern and eastern Africa in applied conservation research. JM is a white female cisgendered non veterinary author with experience working in veterinary associated fields. She identifies mainly with a socio-constructionist epistemological position.

The methods were reported in line with the consolidated criteria for reporting qualitative study guidelines developed by Tong et al. (31) to provide a transparent comprehensive report. Per Braun and Clarke and Tong et al. quotations were used to support the research position (32).

### 2.2. Data collection

Purposive and snowball sampling was used to identify and select participants. Participants were selected if they managed a fully operational conservation dog program in Africa or were training dogs for deployment to conservation dog programs in Africa. An online search engine and professional contacts were used to find the programs. Participants were contacted through their email address or contact page with the same email template (See Supplementary material). Participants also informed the interviewer of other programs that were not originally found through an online search, and these programs were contacted through their contact page on their website, email address published by their website or through informal introduction by the participant. Prior to the interview, participants received an email outlining the aims of the interview, the consent and withdrawal process (See Supplementary material). Interviews were conducted face to face or through an online call between 12th November 2019 to 30th March 2020. Fifteen interviews were conducted from fourteen different conservation dog programs. There were ten heads of program, three trainers, two handlers and two veterinary surgeons. More than one participant was interviewed per organization if it was deemed appropriate by the participant and interviewer for acquiring the most detailed information.

Semi-structured interviews using an interview guide were conducted. The interview guide was piloted before use with colleagues with experience in the field. The topics included in the interviews covered the structure and purpose of the organization, day to day work and care of the dogs, routine healthcare, health problems encountered including mortalities, infectious disease and non-infectious disease, the availability of veterinary and laboratory skills and facilities, and facilities or competences desired by the program to improve their conservation dog’s health (Table 1). The interview was conducted by a 32-year-old female white British veterinary surgeon (NEE), with no other people present at the interviews. The interviewer had had previous non-professional contact with two of the participants (CD8 and CD9b). The interviews lasted an average of 69 min (±21.5 min) and ended when all the available information was gathered. The interviewers were also invited to share photographs and further information following the interview. The iterative nature of the qualitative research interview meant that the focus of some questions altered as more was learnt about the subject. For example, the factors affecting the dog-handler relationship were discussed in much more depth as its importance was brought into focus.

**Table 1:** Main interview topics covered (see Supplementary material for the full Interview Guide).

Program structure	Number of dogs
	Breeds
	Nature of work
Husbandry	Housing and feeding
	Transport facilities
	Husbandry skills
	Working/training hours
	Vaccination protocols
	Parasite control protocols
	Dental care regime
	Health screening
	Record keeping
Health problems encountered	Most common health problem
	Most serious health problem
	Infectious disease
	Trauma
	Heat exhaustion
	Poisoning
	Gastrointestinal disorders
	Behavioral disorders
	Chronic conditions
	Unidentified disease
	Main causes of mortality/retirement
Conflict encountered	Dog-wildlife conflict
	Dog-human conflict
Veterinary facilities	Veterinary facilities available
	Laboratory facilities
	Accessibility to veterinary and laboratory facilities
	Veterinary capabilities of staff
Areas that would benefit from further training or investment in facilities	Husbandry facilities
	Husbandry skills
	Veterinary facilities
	Veterinary skills
Anything that is particularly important	

The interviews were recorded with Otter.ai software (AI Sense Inc. 2019) transcribed verbatim by the interviewer with additional information attached to each transcription document. The transcript documents were sent back to the participant for comment, nine participants responded to the invitation to comment on transcripts with few or no comments and five did not respond. The results were disseminated to the participants in the form of short, easy to read summaries.

### 2.3. Data analysis

The transcriptions were coded by the interviewer using NVivo12 software and themes were generated from the codes and then refined (32, 33). The themes were sent to the co-authors for sense checking before and after refinement. NEE was primary analyzer- from the position of an informed perspective as a veterinarian with an understanding of the literature. Per Braun and Clarke a theoretical approach was taken to the taken with engagement with the literature prior to analysis. In this way authors brough *a priori* ideas to their interpretation of the themes (32).

### 2.4. Ethics statement

The project was approved by The University of Edinburgh Human Ethical Review Committee (Reference HERC_410_19). To gain approval, all efforts were made to minimize harm to participants sharing potentially sensitive information about their activities with the working dogs and the welfare challenges that were in place. Participants were anonymized and individually identifiable information was withheld. Informed consent was required before recording the interviews. Participants were informed that although every effort was made to make them identifiable was made, they may still be identified due to the small number of conservation dog programs. Participants could withdraw from the study at any time and the contact details of the first author were made readily available.

## 3. Results

### 3.1. Overview of participants

The overall structure of the programs that participants represented is summarized in Table 2. Thirty-two potential participants were contacted. Fourteen organizations in total were interviewed (44%), with remaining organizations either not responding (31%), initially responding but without further contact (19%) or not having a conservation dog program running at that time (6%). All organizations interviewed were in southern and eastern Africa. Fourteen interviews involved only one participant, and only one interview per organization was conducted apart from CD10 whereby the handler and veterinary surgeon were interviewed separately for logistical reasons.

**Table 2:** Summary of participants.

Program attribute	Summary
Length of program	Range 3–31 years
	Median 7 years
Number of dogs	Range 30–40 dogs for training programs
	Mean 4 dogs for conservation organizations
Breeds	Range Belgian Malinois, bloodhound, Labrador retriever, pointers, Hanoverian hound, German shepherd, spaniel, Weimaraner, medium crossbreeds.
	Mode Belgian Malinois
Age of acquisition	64% acquire adults, 22% puppies, 14% a combination.
Health check	57% preadmission health checks, 36% no health check, 7% ensured health of parents
Type of work	44% detection and tracking,
	14% species detection,
	14% tracking only,
	14% detection, tracking and apprehension,
	7% tracking and apprehension
	7% detection only

### 3.2. Thematic analysis

Five major themes were generated from the coded data, three affective themes which influence how participants responded to the challenges associated with this kind of work and characterized their motivations and feelings in the role. The final two themes related to the difficulties in managing these programs and how veterinary support interacts with the management choices being made. The themes are:

1) Strength of handler-dog relationship
2) Importance of handler training
3) Acknowledgement of risk to dog
4) Challenges in management
5) The relationship with veterinarians

In summary, the challenges in each aspect of the conservation program demonstrate how difficult it can be to have a functioning conservation dog program, resulting in dogs that accumulate high value. From acquisition to training of both handlers and dogs, through to management and then finally retirement, participants spoke of similar challenges along the way (Table 3). Upon overcoming these challenges and then seeding the dogs succeed, handlers formed strong relationships with their conservation dogs, and were highly motivated to support each dog as an individual. This was strengthened by a wide recognition of the dangers to the dog inherent within the work, such as the effect of temperature extremes. Participants viewed handler selection and training as key to this relationship, particularly in supporting dogs in these high-risk roles. Due to the high investment level in the handler and the strength of relationship between the dogs and handlers, a major concern was how to appropriately prevent, diagnose and treat disease in order to obtain the maximum benefit from the handler-dog relationship. Program staff relied on management practices that did not always have a strong evidence base in veterinary medicine. Program staff found it difficult to find and evaluate evidence related to the dogs’ management. In areas with trypanosomiasis, it was a major concern as it there is no way to eliminate the risk of infection, diagnosis was not always feasible in remote locations with no veterinary diagnostic capabilities, treatment was often unsuccessful and if the dog did survive, infection often resulted in long term effects resulting in the requirement for the dog to retire from work. Compounding this was the challenge of establishing a trusting relationship with veterinarians. While a “good veterinarian” was perceived to be highly valuable because of the support that they gave to the valuable conservation dogs, participants that struggled to establish such relationships with veterinarians felt their dogs’ health to be vulnerable and were frustrated by the inadequate care that resulted from the lack of access to a veterinarian that they trusted. Each of these themes reveals something of the challenges in managing conservation dogs in these settings and provides useful information regarding how the veterinary profession can support this unique working dog partnership.

**Table 3:** The challenges of launching and maintaining a conservation dog program.

Aspect of conservation dog program	Challenges
Acquisition	Expense, logistics, loss of asset and waste of resources if dogs are discharged or early or die.
Handler selection and training	Handlers have no prior experience, skill required in selecting handlers.
Dog training	Logistics and cost of initial intense training, maintenance training and corrective training.
Health management	Limited evidence base, difficulty acquiring medications, cost of medications, cost of evacuation, fear about side effects of prophylactic regimes, difficulty finding competent veterinary care, limited access to specialist care, infectious disease, heat stress.
Program management	Skill fade, maintaining handler performance and morale, dog security, deployment to remote sites, evacuation.
Retirement	Logistics of retirement, availability of retirement homes.
Accountability to donors	Pressure to be productive.

#### 3.2.1. Strength of handler-dog relationship

Trainers and heads of programs recognized the importance of fostering the handler-dog bond as handlers were heavily relied upon for managing the health of their dogs and minimizing the risks inherent in the conservation dogs’ work. Overcoming the challenges of having a functioning conservation dog program as well as seeing the dogs succeed created a strong bond between the conservation dog staff and their dogs. This strong bond was demonstrated in one case by the suspension of the program after the accidental deaths of one dog and the lengths that another participant was prepared to go to in caring for his dogs despite budget constraints:

*“But then during one of the training sessions the handler threw the ball quite a […] there was a giant hole and the dog fell headfirst into the hole and broke her back. And she ended up having to be euthanised. And we were borrowing her, we were not even renting her and it was devastating for us to have to contact CD7 [the trainer that the dog was on loan from] and he came up and said that she would not be happy so she ended up having to be euthanised. So then we took a year off, where we were trying to decide if this is something we really wanted to get into.” CD11, Head of programme.*

#### 3.2.2. Importance of handler training

All but three participants communicated that the handlers were central to the health of the conservation dogs although it should be noted that this was not explicitly asked within the interview schedule. Of the three participants that did not communicate this, two were handlers themselves and one was a veterinarian that was not involved in the day-to-day activities with the dogs. Participants described multiple factors resulting in the establishment and maintenance of a team of dedicated handlers selected from a cohort who had had no prior experience of handling dogs. They described the skill that it takes to select a team of handlers and train them to work with the dogs as well as foster a human-dog bond which results in them valuing their dogs as well as noticing subtle changes in their dogs’ behavior that might be indicative of ill health.

*“Because if the handler, I mean the handlers are like the basis of everything. If they are not operating the dogs, the dogs do not operate does not matter how good your dog is. And there’s so many complex factors in there with the human from private stuff to work stuff to everything, you know.” CD12 Head of programme.*

*“I think key to the whole thing is having the right handlers. The handlers make or break them. And we are really fortunate we have got a really good team who really care about it. You have the right handler. A- you get a productive dog; B you know when it comes to welfare, you know they are on it early.” CD13 Head of programme.*

Participants that had a selection and training process that resulted in dedicated handlers saw this as a major benefit for their dogs’ health. The handlers were instructed that the health and welfare of their dogs was their responsibility and of paramount importance. The health of the dogs should not be put at risk, even if the outcome of the work would be compromised. They were trained to recognize, prevent and manage heat stress, prevent dietary indiscretion, perform basic first aid and know their dog’s individual character and thus recognize any health problems. They were trained to perform a health check or groom daily, which superficially served the purpose of removing ticks, checking for thorns and keeping the coat in good condition. On another level it gave handlers the opportunity to recognize early signs of ill-health. One a deeper level it fostered the dog-handler bond that was not necessarily culturally intrinsic in the handlers.

*“And so yeah, so the I think that’s one of the main things is the handler training is that, you know, the dogs, the dogs’ health and welfare, and wellbeing is absolutely number one priority. And without that they do not have a job … there’s just no question that the dogs do not get that temperature taken. There’s no question that the dogs do not get groomed. There’s no question you know, it’s become muscle memory now for such a long period of time that it’s not really questioned..” CD14, Head of programme.*

*“Yeah like we groom every day. You obviously do not need to groom every day, but we do it every day. […]. Other dog guys come to me they are like, other dog people in general. They’re like, that’s a bit excessive, I’m like I absolutely agree with you. […] I have the sequence of grooming- again it’s on my notice board. Everybody does the same thing […]and if you follow the sequence, you identify injuries on the dog, you can find parasites, you can find thorns and whatever. It’s not about the grooming, it’s about the process and maintaining a healthy working dog. So, but it’s also rapport development.” CD5 Head trainer.*

*“So grooming every day. It’s how we keep our dogs healthy to be honest. Spending time with the handler and they do the health check points. We have 10 health check points, and they groom and they have the connection and off they go.” CD7 Head trainer.*

Additionally, participants recognized that without maintenance training there is a risk of skill fade which puts the whole program at risk, including the health of the dogs. Handlers invariably received initial training from experienced trainers, but the frequency of maintenance training whereby trainers returned to the program to deliver further training varied considerably. Infrequent maintenance training was seen to impact the performance of the dog-handler team negatively. CD14 recognized the value in employing a resident experienced trainer:

*“I think other units have missed out on because they get that dip in and out. Somebody will install and then leave and then come back six months later and they fix all the behaviour and all the problems that have evolved during that six-month gap. Get everything right again after a couple of weeks and then they disappear for another six months and you keep getting that skill fade.” CD14 Head of programme.*

CD12 recognized the value in regular visits from an experienced trainer:

*“We have them at minimum, every two and a half to three months maximum four months, there is at least three or four visits a year, they are with us, where they spend two weeks with the handlers and the dogs. Just tightening up the screws and, and then adding every time adding to the learning to the training.” CD12 Head of programme.*

#### 3.2.3. Acknowledgement of risk to the dog

Participants described how they must balance the health risks that come with working and training against the requirement to train and work. High temperatures, high tsetse burden and rough terrain meant that dogs had to cease working or training at times, which could be seen in the short term as a loss of workdays, but without doing so could lead to disease resulting in a loss of many work days and even death. Snake envenomation, snake venom ophthalmia, minor injuries, intoxication and trauma were other threats to the dogs’ health during work.

*“It’s really horrible, and then getting onto later that actually causes one of my main issues or not a main issue but it’s just like, ya when you call like, it’s like an acute health problem, so I get a lot of thorns in the pads, thorns in the bodies, I’ve had to pull the dogs because they cannot work in areas with thorns, it does not even help with booties because it was going into their body, and it’s just not worth me having the dog out of work.” CD3, Handler.*

All participants experience the challenges of working in extremes of temperature and it was taken very seriously. High temperatures dictated training times and even whether the dogs would be taken out at all.

*“Sometimes we do not even take the dogs out. Depending on the temperature. The ground can get so hot you cannot put the dogs on the ground. Part of our training, all our field rangers are trained to feel the ground. It’s not about just the air temp, it’s about the ground temperature and it radiated temperature coming up from the ground.” CD6, Trainer.*

Recognizing when dogs were showing early signs of heat stress and treating it was an important aspect of handler training. This adds to the importance of competent handler training in conservation dog health management and the responsibility placed upon handlers to safeguard their dogs’ health.

*“And I feel like our handlers are very, very in tune with heat. It was drilled into them, in the start of the program, just really intense training on, you know, just animal welfare for the first four months before they did anything else.” CD4, Head of programme.*

Handlers were expected to recognize signs of early heat stress on an individual basis, as it was understood that individual dogs would express symptoms very differently and have varying levels of tolerance.

*NE: “What about heatstroke? […]do you have problems with that?” CD2: “So currently not. I think it’s because when I started with the guys it was a big thing on monitoring of dogs. I was really bad on that, if they did not do it I did not take it [well], and monitoring quite well and rehydrated dogs quite well so I have not had [heatstroke occur] yet. Close to it, yes, but the handlers quickly pick it up and do something about it.” CD2, Head of programme.*

*“When you see a bloodhound tracking and now, once he is not good, he leaves the track and tries to go to the bush. But also if you have the dogs you know, this dog will go certain kilometres you know that a certain dog can go 12 kilometres. But when they are on a real scenario, we give one 8 km, you remove and give another dog. We do not want the dogs to be more tired. Because you still need- when the other dog is tired you remove him again. So we make sure they get a lot of rest, a lot of water.” CD10, Handler.*

Perhaps due to the focus on it during training, no participants reported deaths due to heatstroke. Only one participant described an episode of heatstroke in one of the dogs, discussing how it could be prevented in the future by reinforcing the common message from participants that each dog expressed heat stress in different ways with differing tolerance levels.

*“You know, when this heat stroke happened, we definitely spent a lot of time discussing the symptoms of heat stroke if you are not paying enough attention properly, and it came up very quickly- it was only 10 min of walking away from the camp and not a strong exertion. And we know now it was the heart murmur was more than caused it than anything else so.”…“We find when she is too hot she does not want to drink water, so even when we give her the right amount of breaks she tends to try to ignore you and pretend she’s not hot, but we also tend to stop her when her temperature gets high now.” CD11, Head of programme.*

These themes strongly impacted how participants considered challenges in their work, particularly around seeking out evidence for disease management, and the importance of their relationship with their veterinarian.

#### 3.2.4. Challenges in management

Programs faced numerous challenges in the management of their dogs. This was compounded by the lack of evidence base in methods of mitigating these challenges. The absence of scientific literature on best practice, the difficulty obtaining correct medications and the reliance on other programs’ experiences in informing their own practices led to programs having a weak evidence base behind their practices. The lack of evidence on best practice for the prevention and treatment of trypanosomiasis was the greatest concern for all programs in areas with a tsetse fly burden. Two programs actively avoided working in areas with a tsetse burden, five programs operated in areas with a tsetse burden and all three trainers had deployed dogs to areas with a high tsetse burden. The remaining five programs were not working in a tsetse fly area at all. The five participants that operated in areas with tsetse flies employed multiple preventative methods and expressed frustration at not knowing what methods were most effective.

*“We are all just guessing? And if anybody can get anything, you know, for dogs in Africa with tryps [with a] bit more research, it would be great because then we would all feel- I just think his lack of confidence really anything else? We are just flying by the seat of our pants!” CD14, Head of programme.*

To address this evidence gap, programs employed multiple preventative methods in varying combinations including fly netting on vehicles and kennels, tsetse targets sprayed with insecticide, insecticide sprayed on vehicles, topical insecticide spray or lotions, regular isometamidium injections and screening for disease with daily temperature checks. One organization also performed regular blood smears to screen for disease. This high level of intensity of management required the dedication of trained handlers. No method was deemed most effective in their experience, but a combination of methods gave program managers the most confidence in preventing disease in their dogs.

*“So, we are super fortunate, and the guys take it extremely seriously, the trypanosomiasis that tsetse issue. We are totally riddled with tsetses, our guys do not train and when they see tsetse flies around, they will move location until they get into an area where there aren’t any tsetses, our dogs are treated all the time, they only move in fly proof kennels and boxes.” CD12, Head of programme.*

All organizations (*n* = 14) using isometamidium long-term to prevent disease were concerned about long-term side effects, although it was not clear what these effects may in fact be:

*“They get their prophylaxis for tryps and all that. I hate it, that there is no product out there for dogs. We mix Samorin with saline solution and half goes into each leg, to make it less viscous.” CD5, Head trainer.*

*“We started treating the dogs from the time that they were here with Samorin every 10 weeks. That is for tryps. And there’s not a lot known about it as far as I know, because there’s just not a lot of people doing that because there’s not a lot of dogs in tryps areas, usually and if they; if they are there they go to treat them every 10 weeks and it is also because it’s quite a hard medicine for them. So it’s tough on the system.” CD1, Head of programme.*

Another area where disease management faced challenges and lacked evidence was in its diagnosis. Participants described how they endeavored to find the cause of death and severe illness in their dogs and expressed frustration that this often was impossible. This often led to presumptive diagnoses. Delays in, or absence of veterinary attention due to isolation, poor sampling and inadequate processing techniques were to blame.

*“And the circumstances are not 100% clear. The fact that he was poisoned that is, that is clear but how it happened yeah, did he pick up something by mistake was he wasn’t on purpose we actually have never been able to establish we also could not find out what poison it was. Okay, even though all the contents of the stomach and blood and a lot of other samples are sent to different labs, but they could not decide.” CD1, Head of programme.*

Programs expressed difficulty acquiring medications and some medications were too expensive for programs. This varied according to the country in which the program was based, but resulted in the same approach to solving the issue. Programs were forced to use less expensive and more easily acquired medication with a weaker evidence base behind their efficacy.

*“For keeping parasites out of the kennels, we have a, I mean it’s just spray. But at the end of the day when everyone enters our kennels we spray. It’s a very thin mix of bleach because we cannot get F10 [in this country] it’s a bit difficult, but it’s a weak mixture of bleach spray, so everyone sprays when they get in” CD2, Head of programme.*

Participants admitted that they did not always pursue a diagnosis or have a strong evidence base behind their decisions when treating disease but would follow practices they felt would maximize the chances of recovery.

*“We have not had a confirmed case of tick fever, but there have been times when a dog has been looking down and we have been giving them Berenil. So that will knock on the head tryps and/or tick fever. So there are cases where we have not had a blood test done. We just treat it straight away.” CD13, Head of programme.*

*“But generally the issue [with antivenom] is one the dose and you do not know how much and you are not sure what snake has bitten. So normally in the bush we are not sure, so what we do is this. We give a bucket of dexamethasone, I mean like 5x recommended dose. Our theory is that it slows down the metabolic rate, and anti-shock and anti-inflammatory and all these things. I do not know! The other thing is electric shock, we use a cattle prod. So if we can see where the bite is which can be quite hard, is we wet down the dog and put a muzzle on the poor fellow and we zap them. All I know is that when we do it they do not die.” CD7, Head trainer.*

#### 3.2.5. The relationship with veterinarians

Participants valued the relationship that they had with the veterinarians starting with the screening process at acquisition. Once deployed, they found it challenging to find veterinarians that they trusted to look after their dogs:

*“getting proper vets- there are, but there’s also some vets where you are like geez I do not know if I’m at the right place.” CD2, Head of programme.*

The nine participants that had a good working relationship with their veterinarian(s) valued it highly. Apart from providing treatment, they communicated with their veterinarian(s) remotely, asking for advice and entrusted them to train their handlers in first aid. Programs that lacked a good relationship recognized this as a challenge to their dog’s health as it prevents them seeking veterinary treatment and advice.

*“I’m fortunate enough that if I call the vet now, I can get to my vets right away. I just need to keep my dogs alive up until then. And we have an open line with, with the vets we started a really good relationship with him where, if I phone him.” CD2.*

Participants would go to great lengths obtaining veterinary care that their dogs required including emergency evacuation by aeroplane to veterinary facilities and even breaking the law:

“Anyway, so she got there, she was fine for months. But the vet here took out the ovaries but left a bit. And the uterus got infected and she got a pyometra. And the really cool [vets] are really great and switched on and South Africa is just across the border so they have lots of kit and it’s all good. So they got her better from that, but she had a cyst on the back wall of her vagina, but it was too close to the back wall of her bladder, they were worried if they did anything they would f*** it up. So we needed to refer her to Jo’burg. So getting a dog into SA is a whole serious problem. So I called a dodgy mate and he smuggled her into SA and she reappeared in SA. Got her up there, my guy got her in Jo’burg. So they broke her pelvis to get at it and rewired it.” CD7 Head trainer.

## 4. Discussion

### 4.1. A qualitative study on the health of conservation dogs

This research study gave a detailed understanding of the health challenges faced by conservation dogs in four countries in eastern and southern Africa, and how they are currently managed. The semi-structured interview meant that aspects most important to the participants were focused on, which positivist research approaches would not convey (28). Qualitative analysis provides an integrative approach to understanding veterinary health problems, particularly those that are poorly understood (29). It allows the communication of scientific and non-scientific knowledge that participants rely upon to make decisions. The essentialist framework meant that the data communicated the reality of the situation. For example, preventing heatstroke is a constant battle- this is assumed to be true and constructs the reality of the situation. It may also allow researchers to learn from those immersed in this specialist field as well (34). Hall et al. acknowledged that those involved in training working dogs are not formally involved in research, and may not have scientific explanations for their practices, but have years of experience from which researchers and peers can learn and highlight areas requiring further applied research (35). This study is an example of the use of qualitative research in the acquisition of these experiences.

It must be understood however that this does not communicate the viewpoints of all conservation dog programs in Africa, and other programs may face different challenges according to their geography and program structure. Programs in western and northern African countries, non-English speaking programs and programs that were not contactable by email were notably absent from the study and may have very different experiences. Programs that had historically been in place but had failed were also absent, so challenges contributing to the failure of programs could not be explored. Failing programs may also have been less likely to respond. However, the interview process allowed for participants to discuss their past challenges as well as current ones and describe how the challenges were overcome and did not lead to failure (36).

### 4.2. The handler-dog relationship

The challenges of having a functioning conservation dog program were multifaceted and ran from the initiation of the program, program management through to retirement of the conservation dogs. These challenges increased the inherent value of the dogs and thus placed a great deal of responsibility on the handlers to keep their conservation dog healthy. A strong handler-dog relationship was important in maintaining the health of the dogs demonstrated by the fact that all participants other than the handlers themselves and the veterinarian who was not involved in the day-to-day care of the dogs stated that it was fundamental to the success of the entire program. Handlers understood and knew dogs as individuals, noticing changes in their health at the early stages, thus allowing intervention before disease was severe. A strong relationship meant handlers prevented them from working in dangerous circumstances such as when it was too hot, or the tsetse challenge (and therefore the risk of contracting trypanosomiasis) was too high. Most studies on the relationship between working dogs and their handlers have found dogs to be in an improved welfare state, have less behavioral problems and for humans provide emotional and psychological benefits (37–41).

Handlers cannot thrive from a strong handler-dog relationship alone. For a strong handler-dog relationship, handler welfare must be prioritized alongside conservation dog welfare, taking a One Welfare approach (42). Handlers are responsible for the health and performance of valuable dogs in a challenging environment. This responsibility should be rewarded proportionately to this high expectation that is put upon them. A combination of logistical support and pastoral care is warranted for this group of specialized individuals that work in dangerous and challenging environments, while safeguarding the health of their dogs. Only recently has the welfare of rangers in Africa come to the forefront of public consciousness (43–45). Although conservation dog handlers do not perform the same tasks, comparisons can be made as both are at the front line of conservation. Improving employment conditions and ensuring adequate equipment and training were key recommendations following a survey of 570 rangers in 12 African countries (43, 44). Only one participant alluded to handler welfare when stating the importance of considering the handlers’ welfare state in the handler-dog relationship. Although no other participants discussed this, it does not mean it was not considered important as the interview focus was on the health of the dogs.

The selection and training process was recognized as being key to the maintenance of the dogs’ health. The selection of handlers was often from a cohort that had not had very much experience of working with dogs or of having a relationship with dogs which is commonly recognized in sub-Saharan Africa (46), so required a unique skill to recognize which handlers would be suited to the job. The correlation between handler-dog proficiency and handler personality, and the dog-handler relationship has been previously established (11, 38, 47–50). None of these studies are directly related to the recognition of health problems. In our study, training inexperienced handlers to ensure the optimum health of the dogs involved instructing them on routine health management such as grooming and recognizing signs of ill health and poor working conditions. Handlers were trained to prioritize dog health above performance. The handler-dog bond was allowed to develop by incorporating routines in which the handler-dog bond could be nurtured. This was directly linked to handler-dog team performance as without the dogs being healthy, they could not perform. Organizations wanting a successful conservation dog program must be aware of this vital step in its establishment.

Handler experience was not recognized as being important in the health of the conservation dogs. This is in line with a study examining factors affecting welfare of military dogs in Belgium (38), but in contrast with several studies on wildlife detection programs (11, 51). This highlights the importance of the initial selection and training process, but as most of the programs had not been running for a very long time (median 7 years) it could be that there were not enough experienced handlers for the benefit of their experience to be recognized. Also, there are no studies looking directly at canine health and handler experience, so without looking at this directly one can only speculate from these performance studies that health is incorporated in handler-dog performance. Future studies investigating the link between canine health and handler experience would be beneficial.

### 4.3. Evidence based approach to healthcare

The lack of evidence-based management of the most important health risks to the conservation dogs was a challenge to the health of the conservation dogs. Evidence based decision making is missing or inconsistent in the working dog field (52–54). In this study, lack of research on the management of health conditions specific to conservation dogs in Africa, lack of access to information including from competent veterinarians, a reliance on easily available information from the internet and other programs and a reliance on traditional management techniques all contributed to management that had a weak evidence base. For example, the use of stun guns to treat venomous bites and stings is a practice that has been discredited in the literature but is practiced by well-meaning conservation dog handlers (55). This is often the case when it comes to managing the health and welfare of working dogs, where small numbers and very specific tasks means that there is a lack of scientific research and a reliance on knowledge bases outside of science (12).

Programs were not able to confidently prevent and treat African canine trypanosomiasis due to the lack of research in this area. There was also concern about the long-term side effects of regular isometamidium injections, but there was no information on what these could be. The only studies so far investigating methods of prevention of trypanosomiasis is of a study on French military working dogs who had isometamidium administered every two to 3 months during their deployment to Ivory Coast or Gabon. Dogs deployed had a reduction in incidence of trypanosomiasis from 12.8% (19/148 dogs) to 2% at 0.5 mg/kg dosage (2/202) to 0% at 1.mg/kg dosage. This supports the use of regular isometamidium injections, but this solitary short-term study does not evaluate the possible long term side effects. Studies on the longer-term effects of the drug would enable programs to make better decisions regarding its use. Understanding the pharmacokinetics of the drug would be a step toward this (56). There is also little evidence on how to prevent the acute adverse reactions that were reported. Intravenous administration is an effective treatment for trypanosomiasis in horses and avoids the muscular necrosis from intramuscular administration (56). This route of administration in dogs could be a way of avoiding side effects, which would be possible in an experimental trial but would need training in intravenous catheter placement and accurate dosing to limit potential toxicities (57). Regular screening as a means of detecting infection early, treating early and thereby reducing morbidity was reported to be successful by one of the participants who lived in an area with a high tsetse challenge (in combination with fly repellent strategies). The participant adopted this method following severe acute reactions in one of their conservation dogs and could also be investigated as an alternative to repeated prophylactic administration. The explanation of this method is an example of the knowledge exchange that Hall et al. described as being valuable (35). There is a clear lack of good quality studies investigating the prevention and treatment of canine African trypanosomiasis and further research is a priority.

The recognition, prevention and management of heatstroke has a considerable amount of published research but is deficient in terms of quality and relevance to the lifestyle of working dogs. Circular referencing and a lack of primary research has led to the establishment of guidelines and the implementation of practices that have a weak evidence base for preventing heatstroke in the working dog environment (58). Dogs are said to be suffering from heatstroke if their core temperatures are above 41°C with central nervous system dysfunction (59). However, a range of studies on dogs doing strenuous exercise range from 40 to 42.2°C without any adverse effects (58). Conservation dogs at extreme temperatures may regularly reach these body temperatures without ill effects, so relying on body temperature alone would be inadequate for considering whether dogs can work or not. Assessing changes in demeanor and thus preventing heatstroke and maximizing their working times is just as important if not more so than checking body temperature alone (58). However, if handlers are inexperienced, it may be safest to rely on numerical values rather than the handler’s assessment of demeanor. Further research on the response of conservation dogs to working in high temperatures is required, so that handlers do not have to rely on poor evidence and evidence that is not specific to working dogs. A call for more research on the physiology of military working dogs and the best management methods for dogs working in extreme environments has already been made in this regard by Baker et al., and with the larger population of working dogs and availability of resources, hopefully this will come about. Conservation dog program workers and managers could apply this research if it were to be obtained either on their own or through their veterinarian, but like all good quality research it must be made readily available and not impede those who wish to access it by being behind a paywall (60). In conclusion, preventing and managing heat stress in conservation dogs should be individualized for each program depending on the population of dogs, the type of work and the experience of handlers.

Funding of research into the health of working dogs in conservation should be prioritized. Guidelines on best practice management of health in conservation dogs should be developed. This requires contemporary research to be available to handlers, support staff and veterinarians involved in conservation dog programs. Programs that pursue an evidence-based approach as best they can do well in advancing the health of their dogs (58). Training and improving accessibility of evidence-based protocols that are relevant for conservation dogs to both the staff of conservation dog programs and their veterinarians could improve this. Improving accessibility of evidence-based resources and education in evidence-based veterinary medicine to veterinarians working with conservation dogs. Working toward an American military working model where there are different stages of training in first aid, refresher courses and further training would mean that training could cater to every level of dog handler experience (21). Workshops like that conducted by Save The Rhino in 2018 and 2019, working with their veterinarians in learning first aid, onsite and online training are all ways that handlers could access support and further their skills (61). Providing veterinarians with access to courses in Evidence-based veterinary medicine, courses tailored specifically to treating working dogs and promoting the use of open-source literature will aid veterinarians to best support conservation dogs.

### 4.4. Conclusion

This is the first study examining the health of conservation dogs in Africa. The implementation of a conservation dog program faced multiple challenges from the acquisition of dogs, selection of handlers, training of handlers and dogs, health management, program management through to retirement. The resulting high investment into the dogs means they had high inherent value. Handlers form strong attachments to their dogs at an individual level. This was strengthened by working with the dogs in the challenging environment and seeing them succeed. Conservation dog programs should consider the fostering of the handler-dog attachment as a priority for the health of the dogs.

The high value of the dogs combined with the strong handler dog attachment meant that program staff were highly motivated to prevent, diagnose and treat disease. Program staff that worked with veterinarians valued their relationship highly, but some programs struggled to find appropriate veterinarians to work with. Providing training to veterinarians working with the dogs and using technologies such as telemedicine would improve access to quality veterinary care. Program staff found it difficult to find and evaluate evidence on best management practices, particularly when it came to preventing and treating trypanosomiasis. Further research into the prevention, treatment, and the effects of chemoprophylaxis of trypanosomiasis would go a long way to closing this evidence gap. Allowing access to the resources that result from any research into the health of working dogs in conservation and related fields should be prioritized such that program staff can work toward evidence-based health management.

## Data availability statement

The raw data supporting the conclusions of this article will be made available by the authors, without undue reservation.

## Ethics statement

The studies involving human participants were reviewed and approved by Human Ethical Review Committee at The University of Edinburgh. Written informed consent for participation was not required for this study in accordance with the national legislation and the institutional requirements.

## Author contributions

NE was the principal researcher. NA was the principal project supervisor. JM provided her support with qualitative research approach. MP provided guidance using her expertise in working dogs in conservation in Africa. All authors contributed to the article and approved the submitted version.

## Funding

This research was funded as part of the author’s Masters course.

## Conflict of interest

The authors declare that the research was conducted in the absence of any commercial or financial relationships that could be construed as a potential conflict of interest.

## Publisher’s note

All claims expressed in this article are solely those of the authors and do not necessarily represent those of their affiliated organizations, or those of the publisher, the editors and the reviewers. Any product that may be evaluated in this article, or claim that may be made by its manufacturer, is not guaranteed or endorsed by the publisher.
