# Supplementary material for: The health of working dogs in conservation in Africa
Source: Front Vet Sci. 2023 Jul 18;10:1179278. doi: 10.3389/fvets.2023.1179278 (PMC10390733; doi:10.3389/fvets.2023.1179278)
Supplement: Supplementary file 1 [file Data_Sheet_1.PDF]

Supplementary Material

The Health of Working Dogs in Conservation in Africa

Nicola Earnshaw[*], Neil Anderson, Jill Mackay, Megan Parker

*** Correspondence:** Corresponding Author: nearnsha@ed.ac.uk

1 Invitation email to participants

Dear xxxxx,

I am a veterinary surgeon reading a Masters in Conservation Medicine at The University of Edinburgh. Through my studies I have become aware of the widespread use of working dogs in conservation in Africa. As part of my final year dissertation, I am investigating the health problems encountered by these incredible animals working in very challenging environments, and how different organisations optimise their health and welfare. I hope to use this study as a platform for sharing different organisation's approaches to maintaining the health of their dogs and identify key areas requiring research.

As part of my study, I would like to interview an appropriate member of your organisation about the work your dogs do, the health problems that you encounter and the facilities that are and are not available to you. Whilst I have some specific questions, I would like the interview to be led mostly by yourselves, so that I can get a real feel for the way that your dogs work and the issues that you have with regards to their health in these challenging environments.

I would really appreciate about 30-45 minutes of your time to interview over Skype. The days that I will be conducting these interviews will be on Tuesday from the DATE/MONTH 2019 to 4th February 2020. I will also be able to do some weekends, and of course would be happy to accommodate you as much as possible if you have a specific date and time in mind.

Please let me know if you would be willing to take part, and what date and time would suit you best. If you have any questions, do not hesitate to ask. I will then send you some further information about the interview content so that you can prepare for the interview. You can change your mind about participating at any time.

Thank you so much, Nicola Earnshaw MRCVS.

2 Preparation for the interview document

Preparation for the Interview

What are the health problems in dogs encountered by Africa-based conservation organisations?

This study is being carried out by myself, Nicola Earnshaw as part of my Masters dissertation with The University of Edinburgh. My supervisors are Dr. Neil Anderson of the University of Edinburgh and Dr. Megan Parker, Director of Research at Working Dogs for Conservation.

Thank you so much for agreeing to participate in the interview. The idea behind this interview is for us to help answer the research question "What are the health problems in dogs encountered by Africa-based conservation organisations?". Using this information, we hope to identify areas for future research required to improve the health of these valuable dogs and provide information to current and future organisations on how the health of their dogs may be optimised.

This information is to help you to prepare for the interview, so that you can gather information beforehand to make the most of our time. Don't worry if you don't know the answers to everything, some topics will be more relevant than others depending on where you are based and what work your dogs do, which is part of what we are trying to find out in our research.

Topics to be Covered

- How many dogs you have, what breeds, what purpose they serve.
- Housing and feeding regimen
- Transport facilities
- Working/training hours
- Vaccination protocol
- Antiparasite protocol i.e. worming, flea, tick, fly prevention.
- If you have any dental care regime
- Health screens that are performed on the dogs and how regularly- this may range from things like weight checks and temperature checks to veterinary checks and blood tests.
- What health problems you encounter:
    - Infectious disease
    - Trauma, poisoning
    - Behavioural problems
    - Chronic conditions like osteoarthritis
- What are the main causes of mortality and retirement of the dogs.
- If you have encountered any problems with respect to the dogs' health as a result of being in contact with wildlife, wildlife products or humans and vice versa.
- How you record this information.
- What veterinary facilities you have- from stethoscopes and thermometers to laboratory and treatment facilities.
- What facilities you desire and what you feel are the main barriers to the health of dogs working in conservation.

3 Interview guide

NICOLA EARNSHAW

Health Problems

What is your annual mortality?

How many of your dogs retire each year?

What is the most common health problem you encounter with your dogs?

What is the most serious health problem that you have encountered?

Please indicate what the morbidity rate (how many suffer but recover), mortality rate (how many dogs die) and also retirement rate (how many dogs cannot return to work as a result of their condition).

Infectious Disease

What is your experience with African sleeping sickness in your dogs?
How often, how to diagnose, treatment, what outcome
What is your experience with tick borne disease in your dogs?
How often, how to diagnose, what treatment, what outcome
Do you have experience of other parasitic diseases or infectious diseases in your dogs?
How often, how to diagnose, what treatment, what outcome

Non-Infectious Disease

Have you had problems with poisoning or suspected poisoning in your dogs?
How often, what treatment, what outcome
Can you tell me about any incidences of heat stroke/exhaustion?
How often, what treatment, what outcome
What kind of traumatic events have your dogs experienced?
How often, what treatment, what outcome
Have your dogs suffered from GDVs?
How often, what treatment, what outcome
What about other gastrointestinal problems?
How often, what treatment, what outcome
What sort of dental problems do your dogs experience?
How often, what treatment, what outcome
Do you have times where your dogs suffer from unidentifiable disease?
    Can you tell me more about this?
Are there any other problems that your dogs suffer from?

Human and Wildlife Health

Have you identified any risks that the dogs may pose to human health?
Have you identified any risks that the dogs may pose to wildlife health?
Have you experienced any human or wildlife health problems as a result of injury or disease from the dogs?
What do you do to mitigate any risk that may be posed to human and wildlife health?

Veterinary Skills

What veterinary skills does your team have in place?

What veterinary skills do you think would benefit your dogs?
Do you have an evacuation protocol if one of your dogs require it?

Facilities

What sort of laboratory facilities do you have?
In house/sent out/no access
What sort of laboratory facilities do you think would be beneficial?
In house/sent out/remote

Laboratory Test
What kind of laboratory testing can you do in house?
What kind of laboratory testing can you send out?
What additional laboratory testing would be beneficial?
In house/sent out/remote

Hospital Facilities
What kind of hospital/treatment facilities do you have for your dogs?
In house/other location/no access
What kind of facilities do you think would be beneficial to your dogs?
In house/other location/no access
Is there anything else you would like to add?

Is there anything that you would like to highlight as being particularly important?
